# Achondroplasia Associated with Bilateral Keratoconus

**DOI:** 10.1155/2012/573045

**Published:** 2012-12-04

**Authors:** Ammar M. Al Mahmood, Hind M. Al Katan, Ghada Y. Al Bin Ali, Samar A. Al-Swailem

**Affiliations:** ^1^Division of Anterior Segment, King Khaled Eye Specialist Hospital, Riyadh 11462, Saudi Arabia; ^2^Division of Pathology, King Khaled Eye Specialist Hospital, Riyadh 11462, Saudi Arabia; ^3^Department of Ophthalmology, Bahrain Defence Force Hospital, West Riffa 28743, Bahrain

## Abstract

We report a rare case of bilateral keratoconus in association with achondroplasia. A 26-year-old male, with a known case of achondroplasia, complained of bilateral gradual deterioration in vision for the past few years. Slit lamp biomicroscopy showed bilateral central corneal protrusion and stromal thinning at the apex consistent with keratoconus. a trial of hard contact lens fitting failed to improve VA in the left eye (LE). Right eye (RE) improved to 20/25. The patient underwent penetrating keratoplasty (PKP) in his LE. Twenty-seven months postoperatively, uncorrected visual acuity (UCVA) was 20/30. Ophthalmologists should be aware that patients with achondroplasia who complain of poor vision should be suspected of having keratoconus once other more common conditions are ruled out.

## 1. Introduction

Achondroplasia is a rare genetic disorder which affects the skeletal system. It is the result of increased signal transduction from a mutated fibroblast growth factor Receptor 3 (FGFR3) which causes an abnormality of cartilage formation. This disorder is characterized by frontal bossing, midface hypoplasia, otolaryngeal system dysfunction, and rhizomelic short stature with normal intellect [1]. Reported ophthalmic features associated with achondroplasia include simple microphthalmos [2], Crouzon syndrome [3], telecanthus, exotropia, inferior oblique overaction, angle anomalies [4], Duane retraction syndrome, cone-rod dystrophy [5], and chorioretinal coloboma [6]. We report a rare case of bilateral keratoconus in association with achondroplasia.

## 2. Case Report

**Figure 1:**
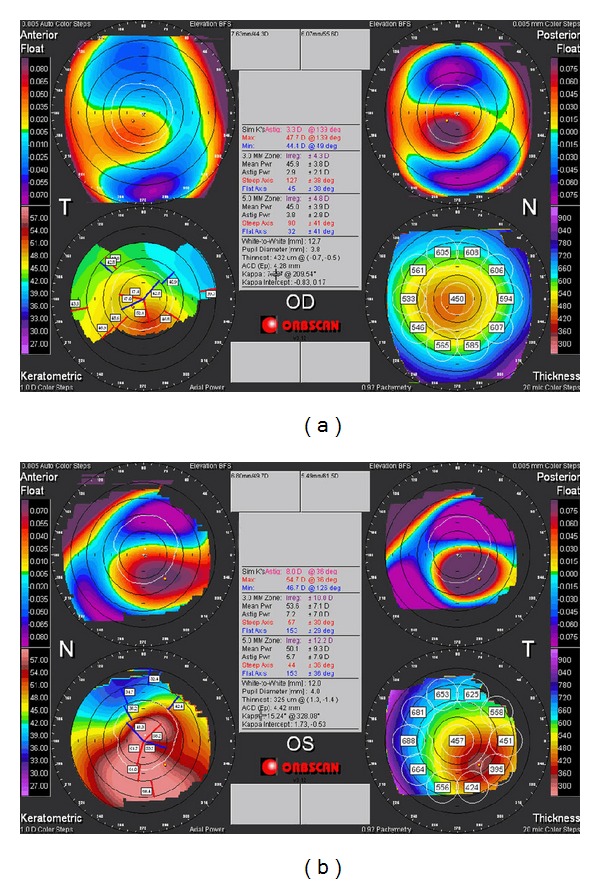
Corneal topography of the right and left eyes showing advanced posterior surface elevation and steepening.

**Figure 2:**
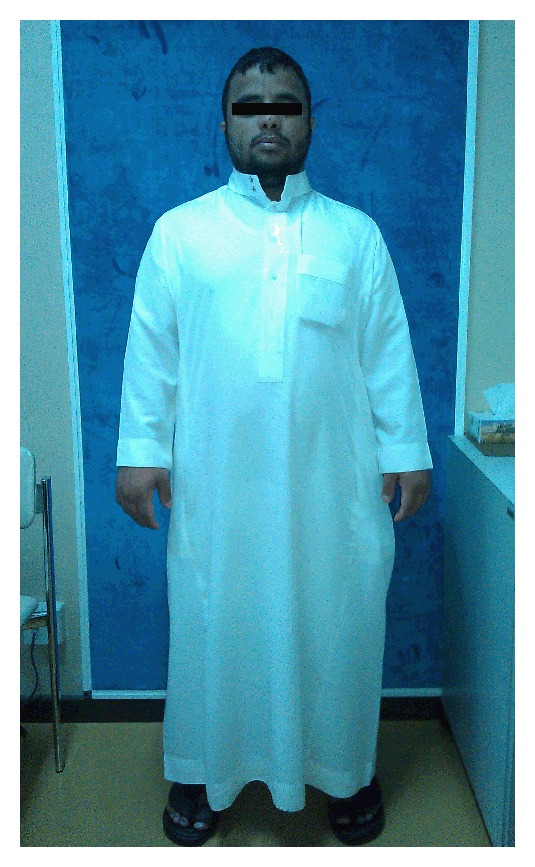
Full body photo of the patient.

**Figure 3:**
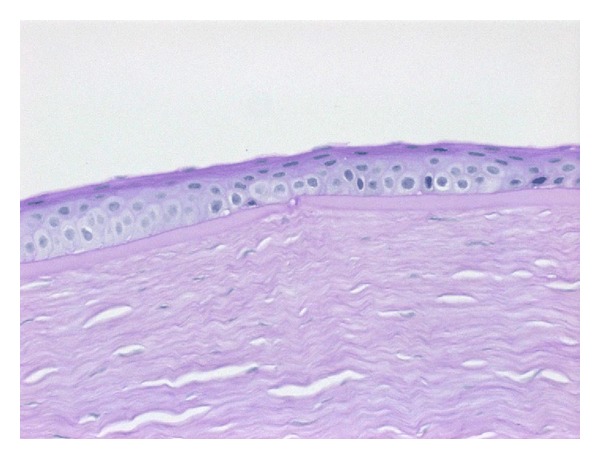
Histopathological section of corneal button illustrating dehiscence in Bowman's membrane (periodic acid Schiff stain, ×400).

A 26-year-old male presented with history of gradual deterioration in vision in both eyes for the past few years. Ophthalmic evaluation revealed uncorrected visual acuity (UCVA) of 20/40 in the right eye (RE) and 20/400 in the left eye (LE) improving with pin hole to 20/30 and 20/50 in the RE and LE, respectively. His refraction was −2.75 + 1.75 × 125 in RE and −22.00 + 7.75 × 70 in LE. Slit lamp biomicroscopy showed bilateral central corneal protrusion and stromal thinning at the apex (Figures 1(a) and 1(b)). Apical corneal scaring was noted in LE. No history of atopy, allergic conjunctivitis, or eye rubbing habitual problem was reported. Achondroplasia was diagnosed based on variable manifestations of the disorder including short stature, frontal bossing, thick fingers, and normal intellect (Figure 2). The patient was the only member in the family of eight siblings with a diagnosis of achondroplasia. a trial of hard contact lens fitting failed to improve VA in LE. RE improved to 20/25. The patient underwent penetrating keratoplasty (PKP) in his LE (Figure 3). Twenty-seven months postoperatively, UCVA was 20/30. 

## 3. Discussion

No previous association between achondroplasia and keratoconus has been previously reported. Such concurrence of achondroplasia and keratoconus raises the possibility of a genetic linkage, although a chance association cannot be excluded. Reports implicate gross structural changes in the gene encoding type II collagen (COL2A1) as the basic defect in achondroplasia [7, 8]. Other reports could not reach the same conclusion [9]. Although type II collagen is not found in the cornea, the presence of a defect in a type of collagen my lead us to think of the possibility that other types of collagen are affected as well. This could explain the association between keratoconus and achondroplasia since corneal stroma contains collagen.

## 4. Conclusion

To the best of our knowledge, no previous association of bilateral keratoconus with achondroplasia was reported. Ophthalmologists should be aware that patients with this syndrome who complain of poor vision should be suspected of having keratoconus once other more common conditions are ruled out.
